# Dataset for efficiency analysis and alliance simulation of listed seafood firms in Vietnam

**DOI:** 10.1016/j.dib.2026.113241

**Published:** 2026-09-10

**Authors:** Nguyen-Nhu-Y Ho, Cong-Thanh Tran, Thi-Hoang-Yen Tran, Khanh-Huyen Vu, Huong-Linh Le

**Affiliations:** International School, Vietnam National University Hanoi, 144 Xuan Thuy Street, Cau Giay Ward, Hanoi, 100000, Vietnam

**Keywords:** Data envelopment analysis, Operational performance, Grey system theory, Aquaculture sector

## Abstract

Strategic alliances have become an increasingly important mechanism for enhancing competitiveness, resource sharing, and value-chain integration in the seafood industry. The dataset presented in this article was developed to support efficiency assessment, forecasting, and alliance simulation among seafood companies listed on the Vietnamese stock market. Financial information was compiled from publicly available audited annual reports of 25 firms covering the period 2020–2024. Six financial indicators, including total assets, cost of goods sold, selling expenses, administrative expenses, net sales, and operating profit, were standardized to construct a harmonized panel dataset. In addition to the original observations, the repository contains forecasted values for 2025 generated using the Grey GM(1,1) model, together with a supplementary dataset of 24 virtual alliance configurations evaluated using the Super-SBM Data Envelopment Analysis (DEA) framework. The data can support future research on efficiency measurement, benchmarking, forecasting, strategic alliances, productivity analysis, and the development of alternative quantitative frameworks for evaluating firm performance.

Specifications Table

**Table:** 

Subject	Business, Management and Decision Sciences
Specific subject area	Management Science and Operations Research, Decision Sciences, Strategy and Management
Type of data	Tables and Figures Raw, Analyzed and Filtered
Data collection	The dataset consists of financial information collected from 25 seafood companies listed on the Vietnamese stock market during 2020–2024. Financial data were extracted from publicly available audited annual financial statements. Six variables were compiled, including total assets, cost of goods sold, selling expenses, administrative expenses, net sales, and operating profit. Firms were included if complete and comparable financial information was available throughout the observation period. The collected data were verified, converted into a uniform unit (million VND), and organized into a standardized panel dataset. Additional forecast and alliance datasets were subsequently generated using the processed financial data.
Data source location	Financial information is publicly available in the audited annual financial statements of seafood companies listed on the Vietnamese stock market. Corporate disclosures and annual reports were retrieved from the official websites of the companies and Vietstock, a financial and securities information portal.
Data accessibility	Repository name: Mendeley Data Data identification number: 10.17632/j98w3r77dh.1 Direct URL to data: https://data.mendeley.com/datasets/j98w3r77dh/1 Instructions for accessing these data: to get these data, go to the direct URL and download the excel data file called “Dataset for efficiency analysis and alliance simulation of listed seafood firms in Vietnam.xlsx”

## 1 Value of the Data

• This dataset provides a standardized panel of financial indicators for listed seafood companies in Vietnam, enabling researchers to examine operational efficiency, productivity, and benchmarking performance within an important export-oriented industry.
• The dataset can be reused to develop, compare, and validate alternative efficiency assessment approaches, including DEA-based models, frontier analysis techniques, and productivity measurement frameworks across firms, industries, or countries.
• The virtual alliance datasets provide a reusable empirical foundation for investigating resource complementarity, cooperative strategies, alliance evaluation, and collaborative performance using quantitative methods and simulation-based approaches.
• By combining historical observations, forecasted indicators, and efficiency assessment datasets within a single repository, the data provide practical insights for managers, investors, industry practitioners, and policymakers to evaluate firm performance, identify potential strategic partnerships, support resource allocation decisions, and enhance competitiveness within the seafood sector.
• Because the virtual alliance dataset was constructed using a simple, fully documented additive rule, researchers are not limited to the specific pairing strategy used in this study (DMU2 combined with each of the remaining 24 firms). The same input–output data and aggregation logic can be applied to construct and evaluate alternative alliance strategies — for example, pairing different focal firms or testing multi-firm alliances — allowing the dataset to support a broader range of partner-selection research beyond the configurations reported here.

## 2 Background

The seafood industry plays a pivotal role in Vietnam’s economy, particularly in export-oriented regions where aquaculture, seafood processing, and international trade constitute major sources of economic activity. As global competition intensifies and international markets increasingly demand higher-quality and value-added seafood products, firms face growing pressure to improve operational efficiency, strengthen supply-chain integration, and enhance competitiveness. In this context, strategic alliances have emerged as an important mechanism for infrastructure sharing, production coordination, and collaborative product development, enabling firms to diversify risks and upgrade their positions within the value chain [1]. Unlike mergers or acquisitions, strategic alliances allow independent firms to create value through resource sharing while maintaining organizational autonomy. Within the seafood industry, such collaborations can facilitate process standardization, improve product quality, optimize operating costs, and support sustainable growth. However, many seafood enterprises remain small- and medium-sized firms that often face constraints in financial resources and reputational capital, limiting their ability to establish effective cooperative arrangements [2].

This study contributes a comprehensive, reusable dataset of listed seafood companies for efficiency analysis and alliance simulation, constructed using an integrated analytical framework that combines Grey System Theory - GM(1,1) and Data Envelopment Analysis (DEA). This hybrid framework simultaneously captures both static and dynamic efficiency, thereby ensuring methodological rigor and practical applicability [3,4]. Specifically, a GM(1,1) Grey forecasting model is applied to the 2020–2024 time series to generate ex-ante estimates for input-output variables in 2025. Based on this framework, the research proposes a partner screening process centered on operational efficiency and resource synergy, offering practical insights specifically tailored to the Vietnamese seafood industry.

Data Envelopment Analysis and its slacks-based variants have become standard tools for efficiency assessment in agriculture, fisheries, and food processing. Applications range from agricultural water-use efficiency in China to environmental efficiency in EU fisheries and Vietnamese rice farming, with Super-SBM increasingly used to rank efficient units precisely and to incorporate undesirable outputs such as emissions [[5], [6], [7]]. Within seafood processing specifically, prior work has shown that efficiency levels vary systematically with product type, with frozen-product processing typically outperforming more complex value-added lines [8].

On the forecasting side, GM(1,1) was selected over classical time-series methods precisely because of its data requirements: models such as ARIMA typically need 50–100 observations and rely on distributional assumptions that a 5-year annual panel cannot satisfy, whereas GM(1,1) is designed for small-sample, poor-information settings and can produce reliable forecasts from as few as four observations [[9], [10], [11]]. Its use of the Accumulating Generation Operator to smooth irregular series and recover an underlying exponential trend has made it a consistently strong performer in short, volatile financial series across domains such as cryptocurrency and emerging-market forecasting [10,12,13].

The Vietnamese seafood sector itself provides the institutional backdrop for this dataset. Since the Doi Moi reforms, Vietnam has grown into a major global seafood exporter, with the sector employing roughly three million people and targeting USD 20 billion in exports by 2030 [14]. Trade agreements such as RCEP and EVFTA have expanded market access [15], while regulatory pressures such as the European Commission's IUU “yellow card” continue to shape firm-level strategy and the sector's push toward value-chain integration and sustainability compliance [16].

Existing datasets on firm efficiency and strategic alliances typically draw on financial and market databases (e.g., Datastream, stock exchange records) or industry-specific technical data, and are used to study how alliances affect productivity, innovation, and absorptive capacity [[17], [18], [19], [20], [21]]. Within this literature, DEA has also been used directly to construct and evaluate virtual alliances: the “virtual DMU” concept — combining the resources of potential partners into a hypothetical merged unit — allows alliance performance to be assessed before any real partnership is formed [Phan & Wang, 2014; Wang et al., 2018]. Hybrid frameworks combining DEA with Grey system theory, fuzzy logic, or AHP have extended this approach to logistics, banking, construction, and pharmaceuticals [20,[22], [23], [24], [25]], providing direct precedent for the virtual-alliance construction method used in this dataset.

## 3 Data Description

The initial population consisted of seafood companies listed on the Vietnamese stock market. To ensure consistency and comparability across the study period, only firms that remained publicly listed and disclosed complete audited financial statements from 2020 to 2024 were retained. Firms were excluded due to missing observations, incomplete reports, or discontinuous financial records. Based on these criteria, a final sample of 25 firms was selected. Table 1 presents the companies included in the dataset together with their corresponding securities symbols and decision-making unit (DMU) codes used throughout the efficiency assessment and alliance simulation procedures. The selected firms represent a broad range of seafood processing, aquaculture, export, and trading activities within the Vietnamese seafood industry. For consistency in subsequent analyses, each company was assigned a unique DMU identifier.

**Table 1:** List of Vietnamese listed seafood companies.

No.	Securities Symbol	Name of Company	Code of DMU
1	CAT	Ca Mau Joint Stock Seafoods Company	DMU1
2	AAM	Mekong Fisheries Joint Stock Company	DMU2
3	ABT	Bentre Aquaproduct Import And Export Joint Stock Company	DMU3
4	ACL	Cuu Long Fish Joint Stock Company	DMU4
5	CMX	Camimex Group Joint Stock Company	DMU5
6	BLF	Bac Lieu Fisheries Joint Stock Company	DMU6
7	KHS	Kien Hung Joint Stock Company	DMU7
8	VHC	Vinh Hoan Corporation	DMU8
9	AGF	Angiang Fisheries Import Export Joint Stock Company	DMU9
10	APT	Saigon Seafood Trading Joint Stock Company	DMU10
11	CCA	Can Tho Import Export Seafood Joint Stock Company	DMU11
12	ICF	Investment Commerce Fisheries Joint Stock Company	DMU12
13	JOS	Minh Hai Export Frozen Seafood Processing Joint Stock Company	DMU13
14	MPC	Minh Phu Seafood Corporation	DMU14
15	SCO	Seaproducts Mechanical Shareholding Company	DMU15
16	SNC	Nam Can Seaproducts Export Import Joint Stock Company	DMU16
17	SPD	Da Nang Seaproducts Import-Export Corporation	DMU17
18	SPH	Hanoi Seaproducts Import Export Joint Stock Corporation	DMU18
19	SJ1	Hung Hau Agricultural Corporation	DMU19
20	FMC	Sao Ta Foods Joint Stock Company	DMU20
21	ANV	Nam Viet Corporation	DMU21
22	IDI	I.D.I International Development and Investment Corporation	DMU22
23	THP	Thuan Phuoc Seafoods and Trading Corporation	DMU23
24	CAD	Cadovimex Seafood Import-Export and Processing Joint Stock Company	DMU24
25	DAT	Travel Investment and Seafood Development Corporation	DMU25

The selection of six variables (four inputs and two outputs) is specifically tailored for Data Envelopment Analysis (DEA) and Super-SBM frameworks, allowing for rigorous benchmarking against existing studies. Financial information was extracted from publicly available audited annual reports and financial statements. All monetary values are reported in million VND. Beyond the primary financial data, a secondary simulated dataset was developed to model virtual alliances between underperforming entities and prospective partners. This approach enables an empirical investigation into resource integration strategies and their impact on the broader seafood industry. The study constructs 24 virtual alliance configurations by pairing DMU2 with each of the remaining 24 firms, then evaluates 49 units in total, including 25 standalone firms and 24 alliance configurations.

Table 2 presents the descriptive statistics of the six financial variables included in the dataset, comprising four input variables (total assets, cost of goods sold, selling expenses, and administrative expenses) and two output variables (net sales and operating profit). The dataset contains 125 firm-year observations covering 25 seafood companies during the 2020–2024 period. All monetary values are reported in million VND. The variables exhibit substantial variation across firms, as reflected in the wide ranges between minimum and maximum values. Operating profit includes both positive and negative observations, indicating differences in financial performance among the sampled companies (Table 3).

**Table 2:** Descriptive statistics of variables.

Variables	Observation	Mean	Standard Deviation	Min	Max
**Inputs:**					
Total Assets	125	1997,090	3047,008	10,975	12,234,180
COGS	125	2004,624	3012,880	8744	13,664,982
Selling Expenses	125	99,700	189,281	40	1351,605
Administrative Expenses	125	50,910	82,159	1940	372,098
**Outputs:**					
Net Sales	125	2288,353	3483,547	14,940	16,425,188
Operating Profit (EBIT)	125	142,869	322,010	48,651	2319,160

**Table 3:** Forecast data of the year 2025 (in million VND).

DMU	Inputs				Outputs	
	Total Assets	COGS	Selling Expenses	Administrative Expenses	Net Sales	Operating Profit
**DMU1**	476,603	477,976	29,546	18,361	559,620	33,605
**DMU2**	208,251	153,533	6534	4825	155,167	3561
**DMU3**	818,818	527,597	36,685	24,084	683,626	150,579
**DMU4**	1818,114	1462,532	142,486	53,550	1794,239	8955
**DMU5**	4352,209	3008,411	85,360	110,849	2991,343	69,423
**DMU6**	575,960	477,593	40,402	19,733	604,058	9977
**DMU7**	461,275	508,279	20,590	14,582	550,115	39,525
**DMU8**	13,959,359	11,377,530	236,041	408,854	13,057,440	1387,857
**DMU9**	224,634	436,689	1129	13,341	498,575	693,057
**DMU10**	180,145	182,088	18,476	15,822	223,092	209,886
**DMU11**	905,526	1359,742	123,231	3397	1449,871	8540
**DMU12**	180,803	24,061	2027	5175	33,383	183
**DMU13**	195,318	37,182	2827	2875	66,566	36,137
**DMU14**	9866,252	12,628,925	750,387	299,009	13,330,469	592,935
**DMU15**	11,261	14,879	2637	3293	24,372	3589
**DMU16**	180,536	333,366	16,953	19,665	395,427	21,982
**DMU17**	382,799	462,343	15,597	36,903	682,998	10,693
**DMU18**	96,194	110,580	6974	39,529	77,325	38,848
**DMU19**	1480,403	1669,153	18,108	29,369	1703,046	50,452
**DMU20**	5663,077	4333,314	90,792	244,949	4874,593	236,259
**DMU21**	4979,292	4999,826	237,545	95,658	5420,630	124,779
**DMU22**	9854,122	7325,771	184,416	113,514	7872,479	98,966
**DMU23**	1400,509	2943,624	134,399	43,956	3192,873	19,360
**DMU24**	9200	16,555	89	3432	15,010	87,140
**DMU25**	1544,853	3185,012	6908	12,549	3285,230	81,381

The complete dataset is stored in an Excel workbook deposited in the Mendeley Data repository and consists of 13 worksheets. The first five worksheets (“Financial Data_2020” to “Financial Data_2024”) contain the original financial information of 25 listed seafood firms, including total assets, cost of goods sold, selling expenses, administrative expenses, net sales, and operating profit. The “Descriptive Statistics” and “Pearson Correlation” worksheets provide summary statistics and correlation matrices for the selected variables. Forecasted financial indicators for 2025 generated using the Grey GM(1,1) model and their corresponding accuracy measures are reported in the “Forecasting Data_2025” and “MAPE_2025” worksheets, respectively. The “Super-SBM DEA_2024 (Before Alliance)” worksheet presents efficiency scores and rankings for standalone firms, while the “Super-SBM DEA_2024 (Virtual Alliance)” and “Super-SBM DEA_2025 (Virtual Alliance)” worksheets contain efficiency assessment results for the simulated alliance configurations based on observed and forecasted data. Finally, the “Alliance Performance Summary” worksheet consolidates alliance rankings and classifications, providing a comprehensive overview of the comparative performance of the virtual alliances.

Financial data were collected from 25 seafood companies listed on the Vietnamese stock market, using each firm's audited annual reports for 2020–2024. Data verification followed a three-person process: one author entered the financial figures from the audited reports, a second author cross-checked the entries against Vietstock, and a third author ran the model to test the resulting dataset before it was finalised. Only firms with complete data for all five years were kept, so the final dataset of 125 firm-year observations has no missing values.

Efficiency scores were calculated using DEA-Solver Pro V13.0, applying the output-oriented Super-SBM model under constant returns to scale (CRS). Total Assets, COGS, Selling Expenses, and Administrative Expenses were specified as inputs, and Net Sales and Operating Profit as outputs, following [3,22,26] and [2,26,27] respectively. The isotonicity assumption between inputs and outputs was confirmed using Pearson correlation prior to running the model (r = 0.481–1.000) [28]. CRS was chosen instead of variable returns to scale (VRS) because super-efficiency scores under VRS can be infeasible for DMUs at the extremes of the input–output distribution, a known limitation of VRS super-efficiency models, whereas CRS super-efficiency scores remain feasible for all DMUs. Given the wide range of firm sizes in the sample (Total Assets ranging from 10,975 to 12,234,180 million VND), this choice ensured that a complete ranking could be obtained for all 49 units evaluated. As discussed in the Limitations section, this means part of the measured inefficiency may reflect differences in scale rather than purely operational performance.

## 4 Experimental Design, Materials and Methods

Financial statements of the selected seafood companies were first collected from publicly available audited annual reports. Data on the financial indicators were then extracted individually and collated into a unified dataset covering the period 2020–2024. Subsequently, for the operational efficiency analysis using Data Envelopment Analysis (DEA), the selected variables were divided into two categories: input measures and output measures. Input and output variables were selected based on production theory and validated using Pearson correlation analysis [2,29]. Descriptive statistics, correlation analysis, and DEA efficiency assessments were performed using DEA Solver Pro 13.0, while GM(1,1) forecasting and MAPE validation were conducted in Microsoft Excel [29,30]. The resulting datasets include historical financial data, forecasted values for 2025, DEA efficiency scores, and virtual alliance configurations.

**Fig. 1:**
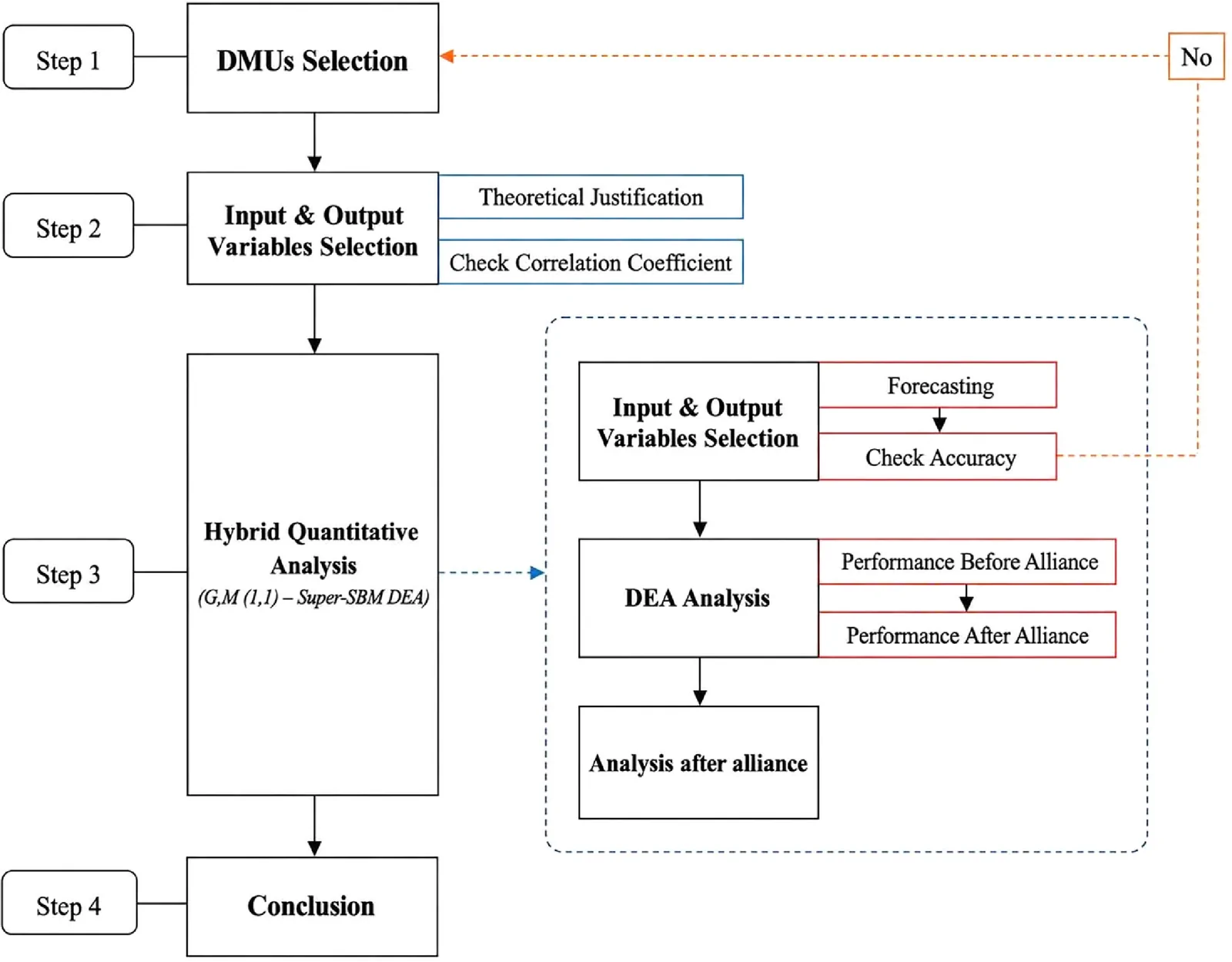
Workflow of dataset construction.

Fig. 1 shows the four steps used to build the dataset, and the three data layers described below follow directly from this process.

Step 1 selects the DMUs. From the seafood companies listed on the Vietnamese stock market, only firms with continuous listing and full audited financial disclosure over 2020–2024 were kept, giving the 25 DMUs used throughout the study.
Step 2 selects the input and output variables. Variables were chosen based on production theory, then checked with Pearson correlation to confirm the isotonicity assumption between inputs and outputs, using the descriptive statistics and correlation results reported in the “Descriptive Statistics” and “Pearson Correlation” worksheets. If the correlation results did not support the isotonicity assumption, the DMU sample was revisited (Step 1) and the process repeated, as shown by the feedback loop in Fig. 1.
Step 3 runs the hybrid quantitative analysis. GM(1,1) is applied to the 2020–2024 observed data to forecast 2025 values, with accuracy checked using MAPE. Super-SBM DEA is then run twice: once on the 25 standalone DMUs to get the before-alliance scores, and once on the 49-unit pool (25 standalone DMUs plus 24 virtual alliances) to get the after-alliance scores, under both observed 2024 data and forecasted 2025 data.
Step 4 summarises the DEA rankings into the Good/Potential/Bad alliance classification and draws the study's conclusions.

Forecasted 2025 values were generated using the Grey GM(1,1) model [12], a first-order grey forecasting technique suited to short annual time series (n ≥ 4). The model was implemented in Microsoft Excel following the standard AGO–least-squares estimation procedure described in [[29], [30], [31]]; the full derivation and a worked calculation are provided in the Supplementary Method file deposited with this dataset. Operational efficiency was assessed using the Super-SBM DEA model [32,33], which extends the slacks-based measure to allow super-efficiency ranking of efficient DMUs (scores ≥ 1); the full model specification and computational settings are provided in the same Supplementary Method file.

To evaluate the operational efficiency of seafood firms and identify potential candidates for alliance formation, this study employs the Super-SBM DEA model. The SBM approach incorporates input and output slacks directly into the efficiency score, providing a more comprehensive assessment of inefficiencies than traditional DEA models [13]. The Super-SBM extension further enables the ranking of efficient DMUs by excluding the evaluated unit from the reference set, allowing efficiency scores to exceed 1 [32]. For a set of (n) DMUs characterized by (m) inputs and (s) outputs, the efficiency of DMU (k) is defined as follows:

(1) $$\rho_{1}=\frac{1-\frac{1}{m}\sum_{i=1}^{m}s_{i}^{-}/x_{ik}}{1+\frac{1}{s}\sum_{i=1}^{s}s_{i}^{+}/y_{ik}}$$

s.t. $x_{\text{ik}}=\sum_{j=1}^{n}x_{\text{ij}}\lambda_{j}+s_{i}^{-},\;i=1,\;\ldots ,\;m$

$$\begin{array}{ccc} y_{rk} & = & \sum_{j=1}^{n}y_{\text{rj}}\lambda_{j}-s_{r}^{+},\;r=1,\;\ldots ,\;s\\ \lambda_{j} & \geq & 0,\;j=1,\;\ldots ,\;n\\ s_{i}^{-} & \geq & 0,\;i=1,\;\ldots ,\;m\\ s_{r}^{+} & \geq & 0,\;r=1,\;\ldots ,\;s \end{array}$$

Here $x_{ij}$and $y_{ij}$ represent the $i^{th}$ input and $r^{th}$ output of DMU j, respectively.

Define $x_{i}^{1}=x_{ik}-s_{i}^{-}$ and $y_{r}^{1}=y_{rk}+s_{r}^{+}$. Accordingly, the SBM model (1) can be reformulated as follows:

(2) $$min\rho_{1}=\frac{\frac{1}{m}\sum_{i=1}^{m}x_{i}^{1}/x_{\text{ik}}}{\frac{1}{s}\sum_{r=1}^{s}y_{r}^{1}/y_{\text{rk}}}$$

s.t. $x_{i}^{1}\geq \sum_{j=1}^{n}x_{\text{ij}}\lambda_{j},\;i=1,\;\ldots ,\;m$

$$y_{r}^{1}\leq \sum_{j=1}^{n}y_{\text{rj}}\lambda_{j},r=1,\;\ldots ,\;s$$

$$\lambda_{j}\geq 0,\;j=1,\;\ldots ,\;n$$

$$x_{ik}\geq x_{i}^{1}\geq 0,\;i=1,\;\ldots ,\;m$$

$$y_{r}^{1}\geq y_{rk},\;r=1,\;\ldots ,s$$

Subsequently, [33] introduced the Super-SBM model to assess the super-efficiency of DMU k as follows:

(3) $$min\rho_{2}=\frac{\frac{1}{m}\sum_{i=1}^{m}x_{i}^{2}/x_{ik}}{\frac{1}{s}\sum_{r=1}^{s}y_{r}^{2}/y_{rk}}$$

s.t. $x_{i}^{2}\geq \sum_{j=1,j\neq k}^{n}x_{\text{ij}}\lambda_{j},i=1,\;\ldots ,m$

$$y_{r}^{2}\leq \sum_{j=1,j\neq k\;}^{n}y_{\text{rj}}\lambda_{j},r=1,\;\ldots ,\;s$$

$$\lambda_{j}\geq 0,j=1,\;\ldots ,n,\;j\neq k$$

$$x_{ik}\leq x_{i}^{2},\;i=1,\ldots ,m$$

$$0\leq y_{r}^{2}\leq y_{rk},\;r=1,\;\ldots ,\;s$$

The optimal value of model (3) is always greater than or equal to 1, ($\rho_{2}*\geq 1$). Notably, the super-efficiency score for DMU k may equal 1 even if DMU k is inefficient. To ascertain whether DMU k is efficient or inefficient, both models (2) and (3) must be utilized. If DMU k is deemed efficient by model (2), model (3) is subsequently applied to compute its super-efficiency. The resulting efficiency scores and rankings are reported in Table 4, which comprises three datasets: (i) the efficiency assessment of 25 standalone seafood firms in 2024 used to identify the focal firm and construct virtual alliances; (ii) the efficiency results of 24 virtual alliance configurations evaluated using observed 2024 data; and (iii) the corresponding virtual alliance configurations assessed using forecasted 2025 data.

**Table 4:** Performance ranking before and virtual alliances.

Rank	Before alliances (in 2024)		Virtual Alliances (in 2024)		Virtual Alliances (in 2025)	
	DMUs	Score	DMUs	Score	DMUs	Score
1	DMU24	7.7375	DMU24	3.5983	DMU24	3.6273
2	DMU9	6.3881	DMU9	2.9489	DMU2_9	2.9024
3	DMU15	4.6191	DMU2_9	2.8468	DMU9	2.7925
4	DMU13	3.5694	DMU10	2.7447	DMU10	2.7284
5	DMU16	2.2989	DMU13	2.5915	DMU2_24	2.5815
6	DMU25	2.1568	DMU2_24	2.4383	DMU2_10	2.4517
7	DMU8	1.8293	DMU2_10	2.2851	DMU13	2.2914
8	DMU22	1.4599	DMU15	2.1837	DMU15	2.2021
9	DMU11	1.3991	DMU2_25	2.0298	DMU2_25	2.0275
10	DMU23	1.2224	DMU25	1.9983	DMU25	2.0043
11	DMU14	1.1927	DMU2_13	1.9787	DMU2_8	1.9539
12	DMU10	1.0703	DMU2_8	1.9277	DMU2_13	1.9108
13	DMU21	1.0064	DMU16	1.8826	DMU2_11	1.8663
14	DMU17	1.0047	DMU2_15	1.8766	DMU16	1.8634
15	DMU4	1.0023	DMU2_11	1.8255	DMU2_15	1.8153
16	DMU6	0.9872	DMU2_14	1.7745	DMU2_14	1.7437
17	DMU3	0.9831	DMU4	1.7234	DMU22	1.7411
18	DMU20	0.9751	DMU22	1.6723	DMU4	1.6791
19	DMU1	0.9749	DMU17	1.6213	DMU17	1.6295
20	DMU19	0.9679	DMU2_22	1.5702	DMU11	1.5559
21	DMU7	0.9653	DMU11	1.5191	DMU2_22	1.4946
22	DMU5	0.9487	DMU2_23	1.4681	DMU2_23	1.4671
23	DMU2	0.8078	DMU14	1.4172	DMU2_17	1.4406
24	DMU18	0.7231	DMU2_17	1.3668	DMU14	1.3848
25	DMU12	0.7181	DMU2_21	1.3149	DMU2_21	1.3122
26			DMU2_16	1.2638	DMU8	1.2862
27			DMU8	1.2128	DMU2_16	1.2143
28			DMU23	1.1617	DMU23	1.1802
29			DMU21	1.1106	DMU21	1.1269
30			DMU2_4	1.0596	DMU2_3	1.1138
31			DMU2_3	0.9985	DMU2_7	1.0518
32			DMU2_7	0.9908	DMU2_4	1.0184
33			DMU2_6	0.9875	DMU2_6	1.0067
34			DMU6	0.9832	DMU6	0.9794
35			DMU3	0.9723	DMU3	0.9653
36			DMU2_20	0.9638	DMU2_20	0.9508
37			DMU2_1	0.9493	DMU2_19	0.9486
38			DMU2_19	0.9387	DMU2_1	0.9352
39			DMU20	0.9076	DMU20	0.9136
40			DMU1	0.9004	DMU1	0.9086
41			DMU19	0.8903	DMU19	0.8779
42			DMU2_5	0.8749	DMU2_5	0.8008
43			DMU7	0.8553	DMU2_18	0.7901
44			DMU2_18	0.8043	DMU7	0.7543
45			DMU5	0.7893	DMU2_12	0.7345
46			DMU2*	0.7398	DMU2*	0.7087
47			DMU2_12	0.7021	DMU5	0.6922
48			DMU18	0.6511	DMU18	0.6391
49			DMU12	0.6038	DMU12	0.6037

Based on the Super-SBM DEA results, DMU2 (Mekong Fisheries Joint Stock Company – AAM) was selected as the focal firm for alliance simulation because it ranked third from the bottom among the 25 DMUs in 2024, representing a relatively low-efficiency but operationally viable firm and providing an appropriate basis for examining potential efficiency improvements through resource complementarity [9,15]. Each virtual alliance $\text{DMU}2_{j}$ (j=1,…,25,j≠2) was constructed by simple, unweighted summation of the corresponding input and output values of DMU2 and $\text{DM}U_{j}$ for the same year: $\text{TA}\left(\text{DMU}2_{j}\right)=\text{TA}\left(\text{DMU}2\right)+\text{TA}\left(\text{DM}U_{j}\right)$, and analogously for COGS, SE, AE, NS, and OP. No weighting, scaling, or normalization was applied prior to summation. These 24 virtual alliances were subsequently evaluated alongside the 25 standalone firms, generating a comparative dataset of 49 DMUs for efficiency assessment. The resulting efficiency scores and rankings before and after alliance formation are presented in Table 4. Based on the ranking results, the virtual alliances were further classified into Good, Potential, and Bad groups according to their relative efficiency performance in 2024 and 2025, facilitating comparison among alternative alliance configurations.

## Limitations

The dataset is subject to several limitations. First, alliance selection and efficiency evaluation are derived from the same set of financial indicators, which may introduce endogeneity in the efficiency assessment process. Second, the GM(1,1) forecasts are generated from relatively short annual time series and should therefore be interpreted as short-term projections rather than long-term predictions. Third, the dataset covers a limited number of publicly listed seafood companies and is restricted to a specific observation period.

## Ethics Statement

The authors certify that the current study does not involve human subjects, animal experiments, or any data gathered from social media platforms, and they have read and complied with the ethical standards for publication in Data in Brief. The primary data is fully authorized for use by the authors.

## CRediT Author Statement

**Nguyen-Nhu-Y Ho:** Conceptualization, Methodology, Software, Data curation, Writing –original draft; **Khanh-Huyen Vu:** Data curation; **Huong-Linh Le:** Data curation, Writing – original draft; **Cong-Thanh Tran:** Data curation, Supervision, Writing – review & editing; **Thi-Hoang-Yen Tran:** Data curation, Supervision, Writing – review & editing.

## Appendix Supplementary materials

Image, application 1
