# Supplementary material for: Dataset for efficiency analysis and alliance simulation of listed seafood firms in Vietnam
Source: Data Brief. 2026 Sep 10;69:113241. doi: 10.1016/j.dib.2026.113241 (PMC13599637; doi:10.1016/j.dib.2026.113241)
Supplement: Supplementary file 1 [file mmc1.docx]

**Supplementary Method**

*Dataset for efficiency analysis and alliance simulation of listed seafood firms in Vietnam*

This document provides the full mathematical derivation of the GM(1,1) grey forecasting model and the Super-SBM data envelopment analysis (DEA) model used to construct the dataset, together with a fully worked numerical example and the DEA-Solver Pro settings used to generate the reported results. This material is referenced in the main manuscript as the "Supplementary Method file."

# **1. GM(1,1) Grey Forecasting Model — Full Derivation**

The GM(1,1) model is a first-order grey forecasting technique designed for short time series with limited information (minimum *n* ≥ 4 observations). Let the non-negative raw data series be

x*^(0)^*(k) = [x*^(0)^*(1), x*^(0)^*(2), …, x*^(0)^*(n)], k = 1, 2, …, n	(1)

## **Step 1 — Accumulated Generating Operation (AGO)**

A new series x*^(1)^* is generated by accumulating the raw series:

x*^(1)^*(k) = Σ*_i=1_^k^* x*^(0)^*(i), k = 1, 2, …, n	(2)

This accumulation smooths random fluctuations in the raw series and reveals an underlying exponential growth or decay trend.

## **Step 2 — Background value (mean generation)**

The background (mean) value of consecutive AGO terms is computed as:

z*^(1)^*(k) = 0.5 · [x*^(1)^*(k) + x*^(1)^*(k − 1)], k = 2, 3, …, n	(3)

## **Step 3 — Least-squares setup**

The GM(1,1) model is defined by the first-order differential equation:

dx*^(1)^*/dt + a·x*^(1)^* = b	(4)

where *a* is the development coefficient and *b* is the grey input (control) term. Its discrete form is:

x*^(0)^*(k) + a · z*^(1)^*(k) = b, k = 2, 3, …, n	(5)

This can be written in matrix form Y = B·θ, where θ = [a, b]ᵀ, and:

B = [ −z*^(1)^*(2), 1 ; −z*^(1)^*(3), 1 ; … ; −z*^(1)^*(n), 1 ], Y = [x*^(0)^*(2), x*^(0)^*(3), …, x*^(0)^*(n)]ᵀ	(6)

## **Step 4 — Parameter estimation**

The parameters a and b are estimated by ordinary least squares:

θ = [a, b]ᵀ = (BᵀB)⁻¹BᵀY	(7)

## **Step 5 — Response (time-response) equation**

Solving the differential equation gives the predicted AGO series:

x̂*^(1)^*(k + 1) = [x*^(0)^*(1) − b/a] · e*^−a·k^* + b/a, k = 0, 1, 2, …	(8)

## **Step 6 — Restored (predicted) values**

The predicted values on the original scale are recovered by inverse accumulation (IAGO):

x̂*^(0)^*(k + 1) = x̂*^(1)^*(k + 1) − x̂*^(1)^*(k)	(9)

Forecast accuracy is assessed using the Mean Absolute Percentage Error (MAPE):

MAPE = (1/n) · Σ | (x*^(0)^*(k) − x̂*^(0)^*(k)) / x*^(0)^*(k) | × 100%	(10)

# **2. Worked Numerical Example — GM(1,1) Forecast for DMU1 (Total Assets)**

The example below reproduces, step by step, the 2025 Total Assets forecast for DMU1 (CAT — Ca Mau Seafood Joint Stock Company) reported in Table 3 of the manuscript (476,603 million VND), so that the calculation can be checked independently.

## Raw data (million VND), 2020–2024

| **2020** | **2021** | **2022** | **2023** | **2024** |
| --- | --- | --- | --- | --- |
| 325,312 | 380,256 | 413,106 | 434,052 | 447,395 |

## Step 1 — AGO series

x⁽¹⁾ = [325,312; 705,568; 1,118,674; 1,552,726; 2,000,121]

## Step 2 — Background values z⁽¹⁾(k)

z⁽¹⁾ = [515,440.0; 912,121.0; 1,335,700.0; 1,776,423.5] (for k = 2, 3, 4, 5)

## Steps 3–4 — Least-squares estimation

Solving θ = (BᵀB)⁻¹BᵀY with the B matrix and Y vector built from the values above gives:

*a* = −0.052587	*b* = 359,019.983

## Step 5 — Response equation

x̂⁽¹⁾(k+1) = (325,312 − 359,019.983 / −0.052587) · e^(0.052587·k) + 359,019.983 / −0.052587

= (325,312 + 6,826,822.9) · e^(0.052587·k) − 6,826,822.9

## Step 6 — Restored values and 2025 forecast

| **Year** | **2020** | **2021** | **2022** | **2023** | **2024 / 2025** |
| --- | --- | --- | --- | --- | --- |
| Actual x⁽⁰⁾ | 325,312 | 380,256 | 413,106 | 434,052 | 447,395 |
| Fitted/forecast x̂⁽⁰⁾ | 325,312 | 386,192.6 | 407,044.8 | 429,023.0 | 452,187.8 / 476,603.5 |

The restored value for k = 6 (year 2025) is **476,603.5 million VND**, which matches the value reported for DMU1 in Table 3 of the manuscript (476,603 million VND; the 0.5 million VND difference is a rounding artifact). The same six-step procedure, applied independently to each of the six variables (TA, COGS, SE, AE, NS, OP) and all 25 DMUs, produces the complete "Forecasting Data_2025" worksheet in the repository.

# **3. Super-SBM DEA Model — Full Specification**

## **3.1 Slacks-Based Measure (SBM)**

For a set of *n* DMUs characterized by *m* inputs and *s* outputs, let x*_ij_* and y*_rj_* denote the *i*-th input and *r*-th output of DMU *j* respectively. The SBM efficiency score of DMU *k* is defined as (Tone, 2001):

ρ = min [ (1/m)Σᵢ(sᵢ⁻/xᵢₖ) ] / [ (1/s)Σᵣ(sᵣ⁺/yᵣₖ) ]	(11)

subject to:

xₖ = Xλ + s⁻, yₖ = Yλ − s⁺, λ ≥ 0, s⁻ ≥ 0, s⁺ ≥ 0

where λ is the vector of intensity variables, and s⁻ and s⁺ are the input and output slack vectors. DMU k is SBM-efficient if and only if ρ = 1 (i.e., s⁻ = 0 and s⁺ = 0).

## **3.2 Super-SBM extension**

To rank efficient DMUs (ρ = 1) among themselves, Fang, Lee, Hwang and Chung (2013), building on Tone (2001), introduced the Super-SBM model, which excludes the evaluated unit k from its own reference set:

δ* = min [ (1/m)Σᵢ(x̄ᵢ/xᵢₖ) ] / [ (1/s)Σᵣ(ȳᵣ/yᵣₖ) ]	(12)

subject to:

x̄ ≥ Σⱼ≠ₖ λⱼxⱼ, ȳ ≤ Σⱼ≠ₖ λⱼyⱼ, x̄ ≥ xₖ, 0 ≤ ȳ ≤ yₖ, λ ≥ 0

Under this formulation, an efficient DMU can obtain a super-efficiency score δ* ≥ 1, allowing a complete ranking of all DMUs, including those that are efficient under the standard SBM model.

## **3.3 Model settings used in this study**

- Software: DEA-Solver Pro, Version 13.0 (Saitech Inc.)
- Model: Super-SBM, non-oriented in the underlying SBM formulation (reported as output-oriented for interpretation of input/output roles)
- Returns to scale: Constant Returns to Scale (CRS)
- Inputs (m = 4): Total Assets, Cost of Goods Sold, Selling Expenses, Administrative Expenses
- Outputs (s = 2): Net Sales, Operating Profit
- Units evaluated: 25 standalone DMUs (before alliance); 49 DMUs — 25 standalone + 24 virtual alliances DMU2_j (after alliance)
- Runs performed: (i) 2024 observed data, standalone DMUs; (ii) 2024 observed data, standalone + virtual alliance DMUs; (iii) 2025 forecasted data, standalone + virtual alliance DMUs

Rationale for CRS over VRS: super-efficiency scores computed under variable returns to scale (VRS) can be infeasible for DMUs at the extremes of the input–output distribution, a known limitation of VRS super-efficiency models. Given the wide range of firm sizes in this sample (Total Assets ranging from 10,975 to 12,234,180 million VND), CRS was adopted to ensure a complete, feasible ranking for all 49 evaluated units.

# **4. Virtual Alliance Construction Formula**

Each virtual alliance DMU2_j (j = 1, …, 25, j ≠ 2) is constructed by simple, unweighted summation of the corresponding input and output values of DMU2 and DMU j for the same year:

TA(DMU2_j) = TA(DMU2) + TA(DMUj)	(13)

and analogously for COGS, SE, AE, NS, and OP. No weighting, scaling, or normalization is applied prior to summation.

## **Worked example — DMU2_9 (2024, observed data)**

DMU2 (AAM), 2024: TA = 202,353; COGS = 149,124; SE = 6,104; AE = 4,772; NS = 153,483; OP = 3,709 (million VND). DMU9 (AGF), 2024: values as reported in the "Financial Data_2024" worksheet. DMU2_9 = DMU2 + DMU9, computed variable by variable, as shown in the "Super-SBM DEA_2024 (Virtual Alliance)" worksheet of the repository.
